# Supplementary material for: On the Chemistry and Physical Properties of Flux and Floating Zone Grown SmB6 Single Crystals
Source: Sci Rep. 2016 Feb 19;6:20860. doi: 10.1038/srep20860 (PMC4759532; doi:10.1038/srep20860)
Supplement: Supplementary Information [file srep20860-s2.pdf]

On the Chemistry and Physical Properties of Flux and Floating Zone Grown SmB_6 Single Crystals

W. A. Phelan,[1,2,3*] S. M. Koohpayeh,[2] P. Cottingham,[1,2,3] J. A. Tutmaher,[1,2,3] J. C. Leiner,[4] M. D. Lumsden,[4] C. M. Lavelle,[5] X. P. Wang,[6] C. Hoffmann,[6] M. A. Siegler,[1] N. Haldolaarachchige,[7] D. P. Young,[7] and T. M. McQueen[1,2,3*]

[1]*Department of Chemistry, Johns Hopkins University, Baltimore, MD 21218, USA*
[2]*Institute for Quantum Matter, Department of Physics and Astronomy, Johns Hopkins University, Baltimore, MD 21218, USA*
[3]*Department of Materials Science and Engineering, Johns Hopkins University, Baltimore, MD 21218, USA*
[4]*Quantum Condensed Matter Division, Oak Ridge National Laboratory, Oak Ridge, TN 37831, USA*
[5]*Applied Nuclear Physics Group, Johns Hopkins University, Applied Physics Laboratory, Laurel, MD 20723, USA*
[6]*Chemical and Engineering Materials Division, Neutron Sciences Directorate, Oak Ridge National Laboratory, Oak Ridge, TN 37831, USA*
[7]*Department of Physics & Astronomy and the Louisiana Consortium for Neutron Scattering, Louisiana State University, Baton Rouge, LA 70803, USA*
[*]*wphelan2@pha.jhu.edu and mcqueen@jhu.edu*

Figure S1. A movie showing the reconstructed X-ray computed tomography (CT) frames of the $^{154}Sm^{11}B_6$ flux-grown crystal. It is obvious that this crystal is composed of two materials, one with a high atomic number (dark contrast) and low Z low atomic number (light contrast). **S4**

Figure S2. A X-ray diffraction histogram obtained laboratory X-ray diffraction. The asterisks and plus signs denote the reflections from aluminum present within the flux grown crystal of SmB_6 and the Si-standard powder, respectively. **S5**

Figure S3. Rietveld refinements to synchrotron X-ray diffraction data at T = 295 K collected on a) cut 2 b) cut 3, and c) cut 4 of the SmB_6 floating zone grown single crystal. The black crosses, red lines, and blue lines correspond to the collected data, refined model, and difference curve respectively. The higher angle data are multiplied by ×10 ($25 \geq 2\Theta \geq 40$) and ×100 ($40 \geq 2\Theta \geq 50$) to highlight the quality of the fit. **S6**

Figure S4. The (110), (310), and (640) reflections for cuts 1-4 where the peak positions were normalized along the x-axis relative to a silicon standard. All peak positions reside at higher angles when going from cuts 1 to 3 for each reflection, showing that the lattice parameters decrease with compositional variations

along the crystal. The overlapping of the (110), (310), and (640) peak positions for cuts 3 and 4 show that these cuts have roughly the same lattice parameters and compositions. **S7**

Figure S5. Concentration (ppm wt) of elements present in the starting material and cuts 1-3 versus Atomic Number. These semi-quantitative trace elemental analyses results were obtained from glow discharge mass spectrometry (GDMS) experiments and are tabulated in Table S1. **S8**

Figure S6. Rietveld refinements to X-ray diffraction data at *T* = 295 K collected on the vaporized material, which amounted only to approximately 1% of the total material from the SmB_6 floating zone single crystal growth. The black crosses, red lines, and blue lines correspond to the collected data, refined model, and difference curve respectively. It is obvious from fits to this data that this vaporized material is a multi-phase mixture of SmB_6 (gray ticks) and SmB_4 (orange ticks). **S9**

Figure S7. A plot of the resistance (R) normalized by the room temperature resistance values ($R_{300\ K}$) versus temperature (*T*) for cuts 1'-3' (open circles) and cuts 1-3 (closed circles, Figure 5) from 0 to 10 K. To check the reproducibility of our resistance measurements, the authors removed the original platinum leads used to collect the data presented in Figure 5 for cuts1-3, polished these three cuts, mounted new leads, and recollected the data (cuts 1'-3'). Additionally, resistances were measured using a new cut between the location of the original cut1 and cut 2 (cut 2'') and a new cut beyond the location of the original cut 4 (cut 4''). Very much like the data for cuts 1-4 in Figure 5, the magnitude of the $R/R_{300\ K}$ and the degree of plateauing decrease and the cut number gets larger for all cuts. Finally, the trend in lattice parameters of 4.1333(4) Å and 4.13284(3) Å for cut 2'' and cut 4'', respectively, agrees well with the resistance and lattice parameter trend for the original cuts 1- 4. **S10**

Table S1. The concentration of differing elements present in the SmB_6 starting material, cut 1, cut 2, and cut3. **S11-13**

Table S2. Crystallographic parameters for the flux grown $^{154}Sm^{11}B_6$ crystal obtained from model fits to the single crystal X-ray diffraction data. **S14**

Table S3. Atomic fractional coordinates, site occupancies, and ADPs for flux grown $^{154}Sm^{11}B_6$ crystal obtained from model fits to the single crystal X-ray diffraction data. **S15**

Table S4. The percent abundance for the differing isotopes of boron and samarium in the flux grown doubly enriched SmB_6 crystal. **S16**

Figure S1. A movie showing the reconstructed X-ray computed tomography (CT) frames of the $^{154}Sm^{11}B_6$ flux-grown crystal. It is obvious that this crystal is composed of two materials, one with a high atomic number (dark contrast) and low Z low atomic number (light contrast).

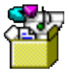

FigureS1.avi

Figure S2. A X-ray diffraction histogram obtained laboratory X-ray diffraction. The asterisks and plus signs denote the reflections from aluminum present within the flux grown crystal of SmB_6 and the Si-standard powder, respectively.

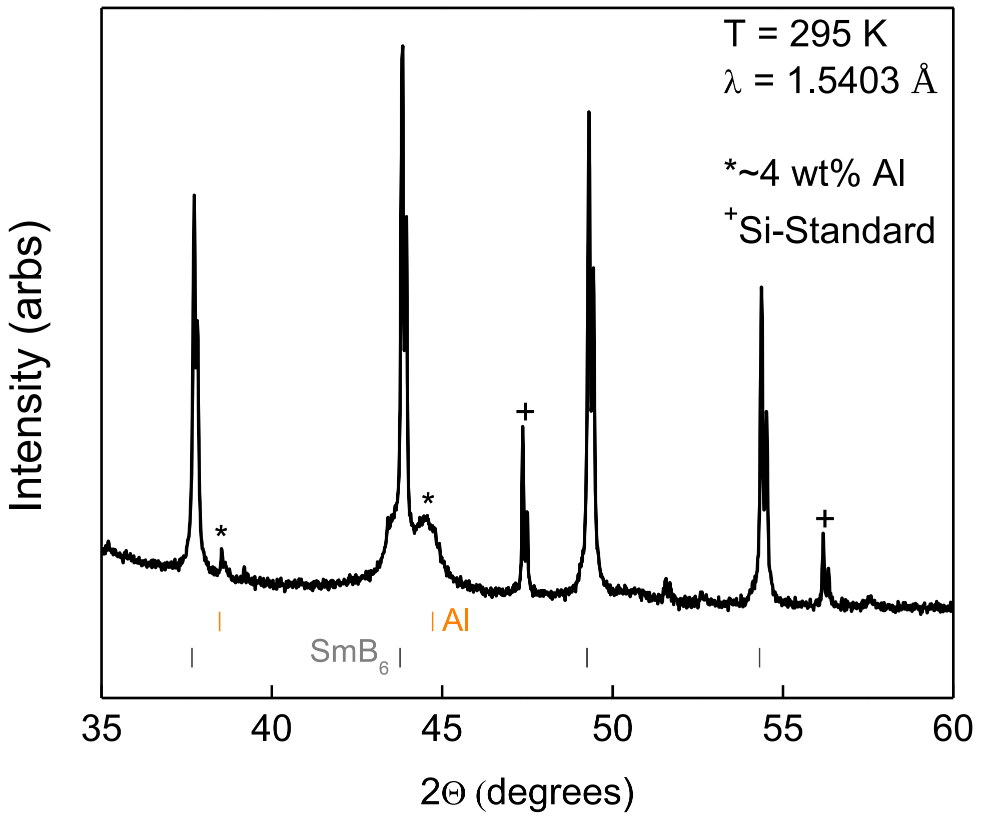

Figure S3. Rietveld refinements to synchrotron X-ray diffraction data at T = 295 K collected on a) cut 2 b) cut 3, and c) cut 4 of the SmB_6 floating zone grown single crystal. The black crosses, red lines, and blue lines correspond to the collected data, refined model, and difference curve respectively. The higher angle data are multiplied by ×10 ($25 \geq 2\Theta \geq 40$) and ×100 ($40 \geq 2\Theta \geq 50$) to highlight the quality of the fit.

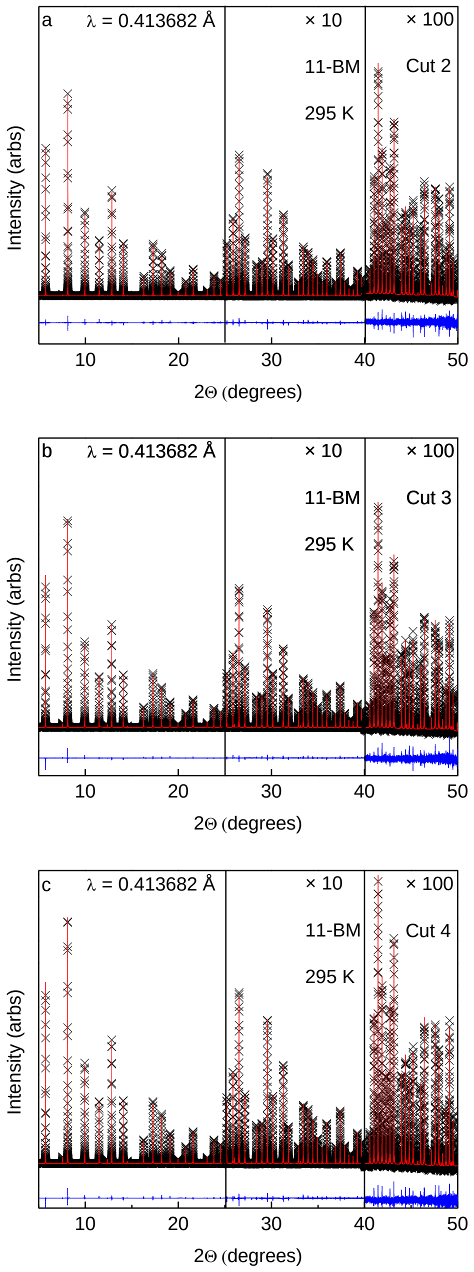

Figure S4. The (110), (310), and (640) reflections for cuts 1-4 where the peak positions were normalized along the x-axis relative to a silicon standard. All peak positions reside at higher angles when going from cuts 1 to 3 for each reflection, showing that the lattice parameters decrease with compositional variations along the crystal. The overlapping of the (110), (310), and (640) peak positions for cuts 3 and 4 show that these cuts have roughly the same lattice parameters and compositions.

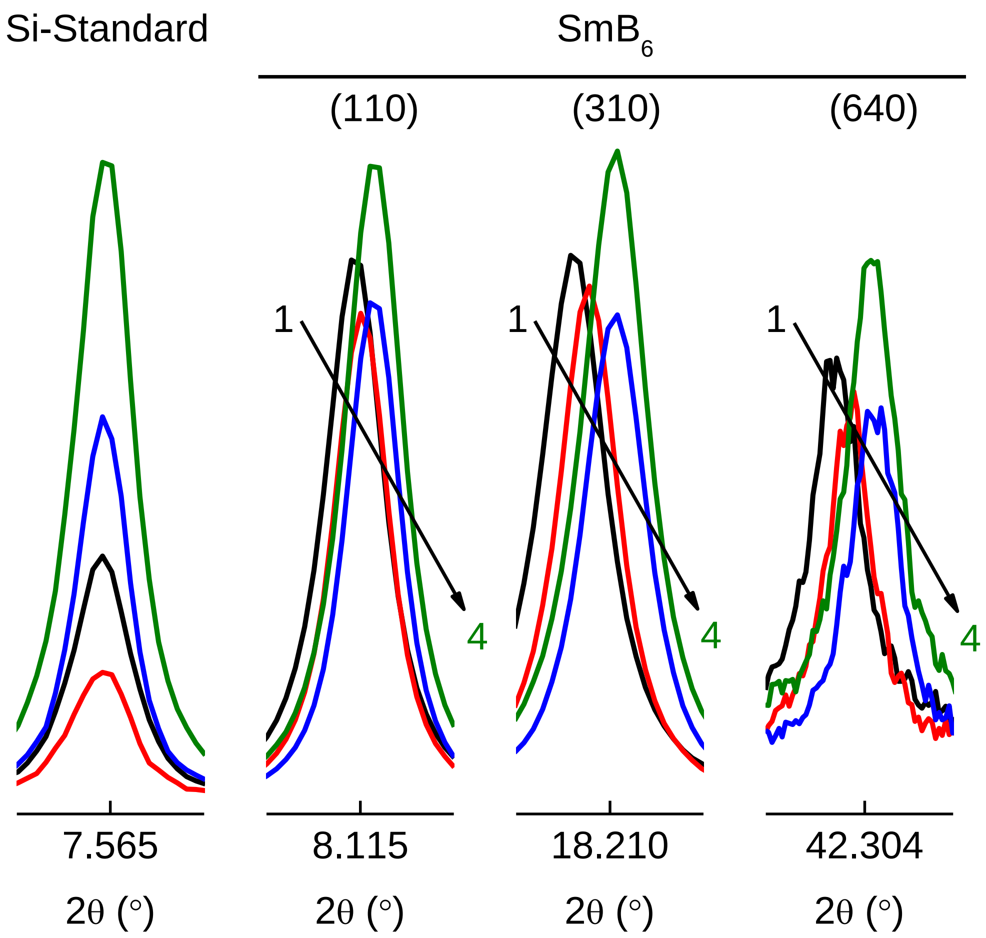

Figure S5. Concentration (ppm wt) of elements present in the starting material and cuts 1-3 versus Atomic Number. These semi-quantitative trace elemental analyses results were obtained from glow discharge mass spectrometry (GDMS) experiments and are tabulated in Table S1.

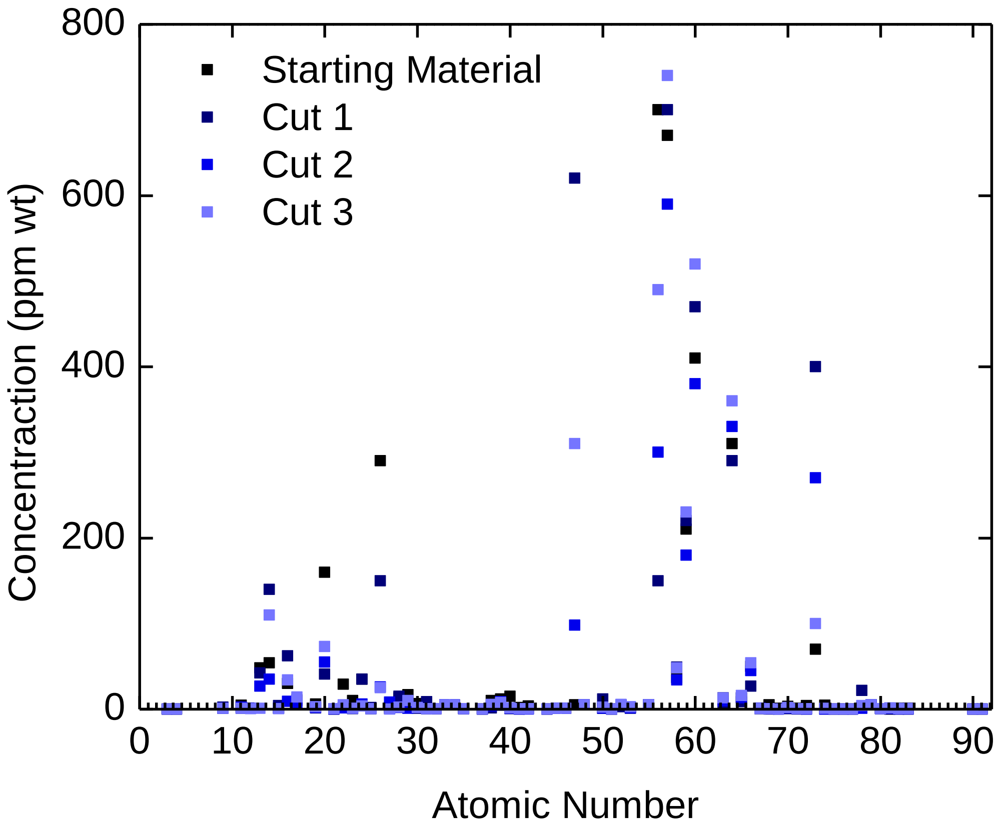

Figure S6. Rietveld refinements to X-ray diffraction data at *T* = 295 K collected on the vaporized material, which amounted only to approximately 1% of the total material from the SmB_6 floating zone single crystal growth. The black crosses, red lines, and blue lines correspond to the collected data, refined model, and difference curve respectively. It is obvious from fits to this data that this vaporized material is a multi-phase mixture of SmB_6 (gray ticks) and SmB_4 (organic ticks).

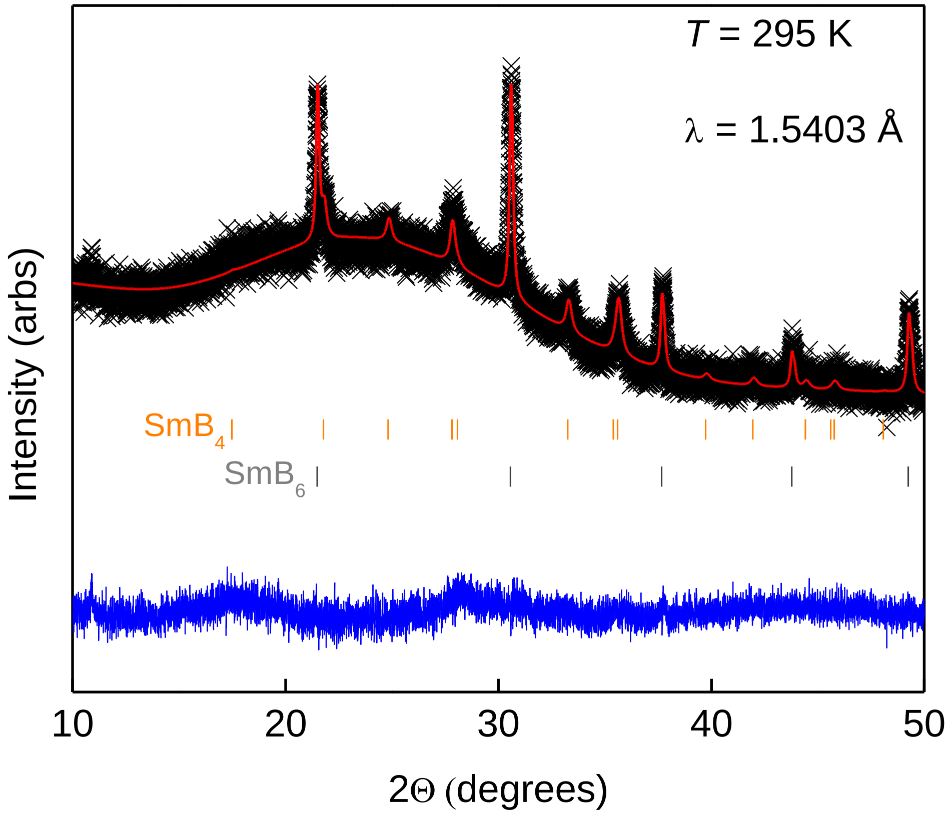

Figure S7. A plot of the resistance (R) normalized by the room temperature resistance values ($R_{300\ K}$) versus temperature (*T*) for cuts 1'-3' (open circles) and cuts 1-3 (closed circles, Figure 5) from 0 to 10 K. To check the reproducibility of our resistance measurements, the authors removed the original platinum leads used to collect the data presented in Figure 5 for cuts1-3, polished these three cuts, mounted new leads, and recollected the data (cuts 1'-3'). Additionally, resistances were measured using a new cut between the location of the original cut1 and cut 2 (cut 2'') and a new cut beyond the location of the original cut 4 (cut 4''). Very much like the data for cuts 1-4 in Figure 5, the magnitude of the $R/R_{300\ K}$ and the degree of plateauing decrease and the cut number gets larger for all cuts. Finally, the trend in lattice parameters of 4.1333(4) Å and 4.13284(3) Å for cut 2'' and cut 4'', respectively, agrees well with the resistance and lattice parameter trend for the original cuts 1- 4.

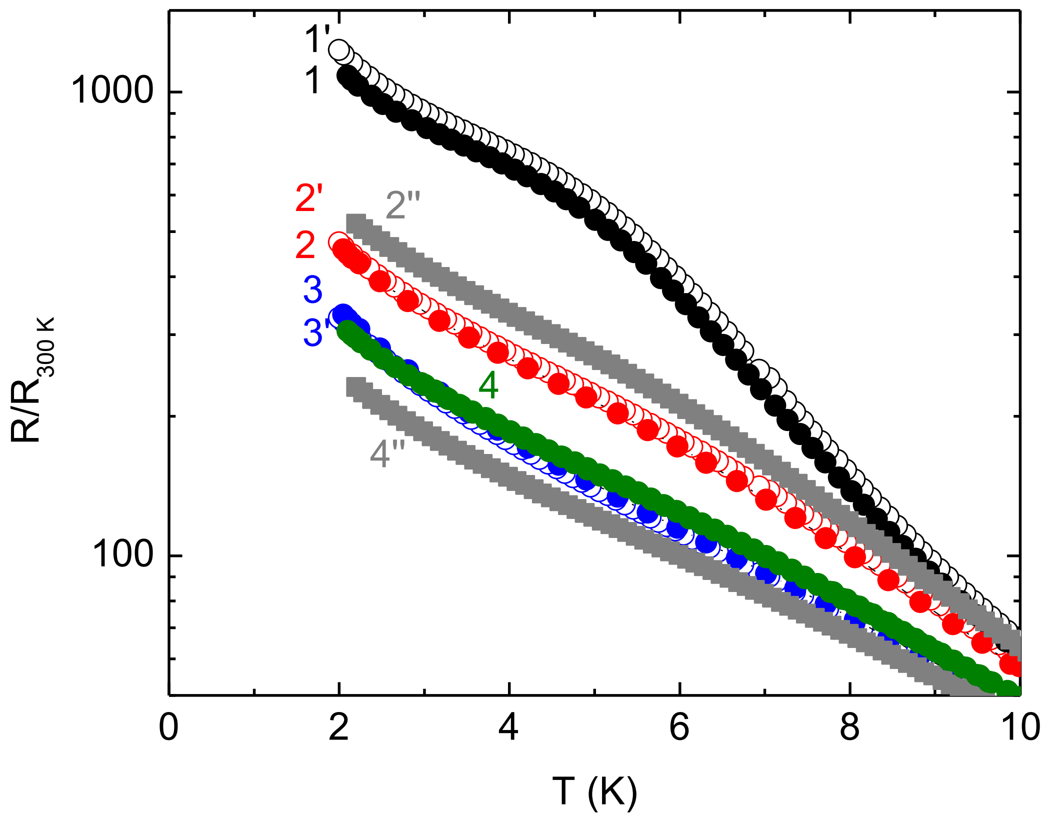

Table S1. The concentration of differing elements present in the SmB_6 starting material, cut 1, cut 2, and cut3.

	Concentration (ppm wt)			
	**Starting Material**	**Cut 1**	**Cut 2**	**Cut 3**
Li	<0.05	<0.05	<0.05	<0.05
Be	<0.05	<0.05	<0.05	<0.05
B	Bulk	Bulk	Bulk	Bulk
F	<1	2.2	<1	<1
Na	4.7	0.97	2.4	0.83
Mg	0.95	1.1	0.91	0.47
Al	48	42	27	0.99
Si	54	140	35	110
P	0.78	4.3	1.1	0.52
S	30	62	9.1	34
Cl	~10	~11	~7	~14
K	6.1	1.3	1.8	2.6
Ca	160	41	55	73
Sc	0.06	0.08	<0.05	0.44
Ti	29	2.0	1.4	5.2
V	10	0.46	0.79	0.76
Cr	35	35	6.0	3.8
Mn	0.65	2.0	0.38	0.27
Fe	290	150	26	25
Co	0.64	2.4	8.2	0.19
Ni	13	15	1.9	2.1
Cu	17	4.3	0.81	9.8
Zn	6.5	0.72	1.9	1.8
Ga	<0.5	8.5	<0.5	<0.5
Ge	<0.5	<0.5	<0.5	<0.5
As	<5	<5	<5	<5
Se	<5	<5	<5	<5
Br	<0.5	<0.5	<0.5	<0.5
Rb	<0.05	<0.05	<0.05	<0.05

Table S1 continued. The concentration of differing elements present in the SmB_6 starting material, cut 1, cut 2, and cut3.

	Concentration (ppm wt)			
	**Starting Material**	**Cut 1**	**Cut 2**	**Cut 3**
Sr	10	1.5	3.8	5.5
Y	12	4.5	8.4	7.7
Zr	15	0.33	0.71	1.2
Nb	1.8	0.10	<0.05	0.29
Mo	3.8	0.45	<0.05	0.49
Ru	<0.05	0.16	<0.05	<0.05
Rh	<1	<1	<1	<1
Pd	<1	<1	<1	<1
Ag	5.2	620	98	310
Cd	<5	<5	<5	<5
In	Binder	Binder	Binder	Binder
Sn	<1	12	<1	2.3
Sb	<0.1	<0.1	<0.1	<0.1
Te	4.2	2.7	4.8	5.4
I	1.8	0.73	1.2	2.9
Cs	<5	<5	<5	<5
Ba	700	150	300	490
La	670	700	590	740
Ce	37	49	34	48
Pr	210	220	180	230
Nd	410	470	380	520
Sm	Bulk	Bulk	Bulk	Bulk
Eu	13	6.6	9.5	13
Gd	310	290	330	360
Tb	13	8.7	13	16
Dy	50	27	45	54
Ho	0.48	0.30	1.1	0.44
Er	5.1	0.15	0.35	0.61
Tm	0.95	<0.05	<0.05	0.11

Table S1 continued. The concentration of differing elements present in the SmB_6 starting material, cut 1, cut 2, and cut3.

	Concentration (ppm wt)			
	**Starting Material**	**Cut 1**	**Cut 2**	**Cut 3**
Yb	3.2	0.35	1.5	1.7
Lu	0.94	0.08	0.25	0.51
Hf	4.1	<0.1	<0.1	0.63
Ta	≤70	≤400	≤270	≤100
W	4.7	<0.1	<0.1	1.0
Re	<0.1	<0.1	<0.1	<0.1
Os	<0.1	<0.1	<0.1	<0.1
Ir	<0.1	<0.1	<0.1	0.19
Pt	1.8	22	1.1	3.9
Au	<5	<5	<5	<5
Hg	<0.5	<0.5	<0.5	<0.5
Tl	<0.01	0.10	0.93	1.5
Pb	<0.05	1.1	0.11	0.28
Bi	<0.05	1.1	0.11	0.24
Th	0.04	0.03	0.04	0.07
U	0.03	0.01	0.02	0.03

Table S2. Crystallographic parameters for the flux grown $^{154}Sm^{11}B_6$ crystal obtained from model fits to the single crystal X-ray diffraction data. The statistical uncertainties are given in parentheses.

Temperature (K)	293(2)
Composition	$SmB_{5.88}$
Space group	*Pm*-3*m*
a (Å)	4.13283(2)
V (Å^3)	70.590(1)
Z	1
Collected Reflections	7089
Independent Reflections	154
GooF	1.18
$R_1[F^2 > 2\sigma(F^2)]^a$	0.009
$wR_2(F^2)^b$	0.020
$\Delta\rho_{max}$ (e Å^{-3})	1.00
$\Delta\rho_{min}$ (e Å^{-3})	-1.36

$^a R_1(F) = \sum ||F_o| - |F_c|| / \sum |F_o|$; $^b wR_2(F^2) = [\Sigma [w (F_o^2 - F_c^2)^2] / \Sigma [w (F_o^2)^2]]^{1/2}$

Table S3. Atomic fractional coordinates, site occupancies, and ADPs for flux grown $^{154}Sm^{11}B_6$ crystal obtained from model fits to the single crystal X-ray diffraction data. The statistical uncertainties are given in parentheses.

T = 293(2) K

atom	Wyckoff Site	x	y	z	Occupancy	U_{11} (Å^2)	U_{22} (Å^2)	U_{33} (Å^2)
Sm1	*1*a	0	0	0	1	0.00776(4)	0.00776(4)	0.00776(4)
B1	6*f*	0.2000(1)	½	½	0.98	0.0036(2)	0.0036(2)	0.00523(14)

Table S4. The percent abundance for the differing isotopes of boron and samarium in the flux grown doubly enriched $^{154}Sm^{11}B_6$ crystal.

	% Abundance of Boron		% Abundance of Samarium						
	^{10}B	^{11}B	^{144}Sm	^{147}Sm	^{148}Sm	^{149}Sm	^{150}Sm	^{152}Sm	^{154}Sm
Doubly Enriched SmB_6 Crystal	3.6(1)	96.4(2.1)	0.0067(2)	0.044(1)	0.041(1)	0.0550(3)	0.0390(2)	0.305(2)	99.51(0.74)
